# The foundations of the human cultural niche

**DOI:** 10.1038/ncomms9398

**Published:** 2015-09-24

**Authors:** Maxime Derex, Robert Boyd

**Affiliations:** 1 Institute of Human Origins, Arizona State University, Tempe, Arizona 85287, USA; 2 School of Human Evolution and Social Change, Arizona State University, Tempe, Arizona 85287, USA

## Abstract

Technological innovations have allowed humans to settle in habitats for which they are poorly suited biologically. However, our understanding of how humans produce complex technologies is limited. We used a computer-based experiment, involving humans and learning bots, to investigate how reasoning abilities, social learning mechanisms and population structure affect the production of virtual artefacts. We found that humans' reasoning abilities play an important role in the production of innovations, but that groups of individuals are able to produce artefacts that are more complex than any isolated individual can produce during the same amount of time. We show that this group-level ability to produce complex innovations is maximized when social information is easy to acquire and when individuals are organized into large and partially connected populations. These results suggest that the transition to behavioural modernity could have been triggered by a change in ancestral between-group interaction patterns.

## Abstract (web summary)

Our understanding of how humans produce complex technologies is limited. Here, the authors use a computer-based experiment to show that the production of complex innovations results from a population process that relies on efficient social learning mechanisms and specific population structures.

## Graphical abstract

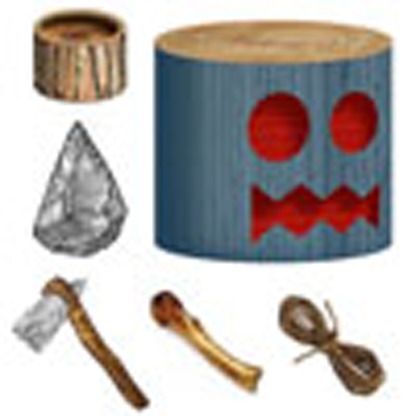

Technological innovations have allowed humans to settle in habitats for which they are poorly suited biologically^1^,^2^. The obvious evolutionary advantage of the ability to make such innovations raises many questions about how complex technologies arise, and why only humans are able to produce them. Many authors have linked our remarkable ability to develop new technologies with our no less remarkable reasoning abilities^3^. The fossil record reveals increasing encephalization in the human lineage during the past 2 Myr ago^4^ and comparative studies demonstrate that brain volume and intelligence covary^5^,^6^, suggesting that modern humans developed complex technology because biological evolution made them smarter than non-humans species. However, (1) the archaeological record indicates that major increases in technological complexity occurred significantly after the appearance of anatomically modern humans^7^, and (2) human populations typically overcome environmental challenges that isolated individuals cannot cope with^1^, suggesting that increased intelligence does not fully explain this phenomenon.

An alternative explanation is that human social learning abilities are also essential for the ecological success of our species^1^. According to this view, humans possess unique social learning mechanisms that stabilize cultural knowledge and allow successive innovations to be gradually added to existing cultural traits^8^. Bursts of cultural complexity that occurred during the Upper Palaeolithic transition (45,000 years ago) could then be the product of demographic processes: bigger populations, that host a greater number of particularly skilful social learners, are more likely to maintain and improve complex cultural traits^9^,^10^,^11^,^12^,^13^.

Although experimental studies have recently confirmed that decreases in social learning ability and population size can both prevent transmission of cultural knowledge^12^,^14^,^15^, a clear demonstration of cumulative cultural evolution in controlled conditions is lacking. Several cultural evolution experiments have shown that the use of social information increases the average performance of individuals^14^,^15^,^16^ and allows increasingly effective artefacts to arise across successive generations^17^, but these experiments have not clearly demonstrated cultural accumulation of technologies beyond what most individuals could accomplish on their own^18^. As a consequence, we should be cautious in drawing conclusions from these experiments about how new cognitive abilities or increased population densities allow populations to succeed where isolated individuals failed.

Here we present results from a large scale experiment involving both human participants and automated learning bots (with and without social learning abilities). Using a combinatorial computer game in which participants had to discover increasingly complex and nested innovations to produce virtual ‘totem poles' (hereafter ‘totems', Fig. 1), we investigated the achievements of (1) isolated individuals and groups of six humans provided with full social information (full treatment, Fig. 2); (2) automated learning ‘bots' (either alone or in groups of six) generating only random variation (reasoning ability treatments); (3) human participants provided with information about the tools made by other group members, but not about the underlying production process (partial information treatment); and (4) participants provided with full social information, organized in groups of three (small group treatment) or in metapopulations of 3 × 2 players (partial connectivity treatment). We find that human reasoning abilities (defined as the ability to produce guided variation) play an important role in the production of innovations, but that subjects working in groups are able to produce more complex artefacts than single individuals working for the same amount of time. We show that this cultural accumulation process is enhanced by learning mechanisms that make social information easy to acquire and large population size. Surprisingly, we also find that the rate of cultural accumulation is higher within partially connected groups than in fully connected groups of the same total size, which suggests that the transition to behavioural modernity could have been triggered by changes in ancestral between-group interaction patterns.

## Results

### Isolated individuals versus groups

**Figure 1:**
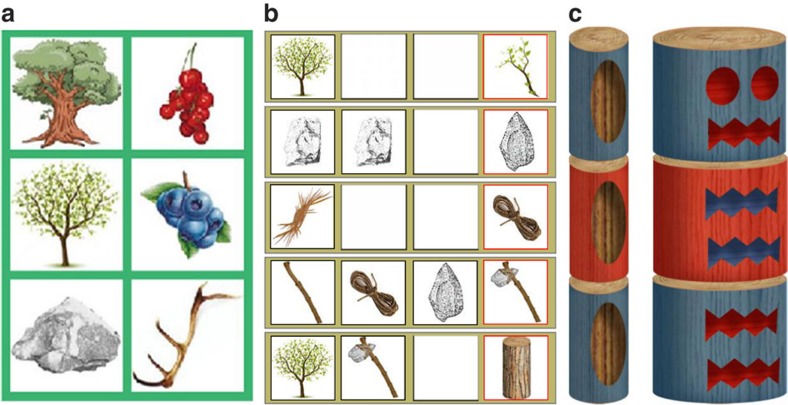
General principle. Our game simulates the real-world innovation process in which the production of complex artefacts (that is, virtual ‘totem poles') depends on the discovering of high-level innovations (for example, axes), whose discovery is contingent on the discovering of lower-level innovations (for example, stone tools), both low- and high-level innovations resulting from a specific production process. (**a**) The ‘resources panel'. Players were provided six initial basic resources that could be combined using a workshop panel containing four slots (Fig. 2). (**b**) Examples of successful combinations. By placing items into a four-slot workshop panel (black squares; only three are depicted here), participants could produce innovations (red squares). Innovations occurred when players produced a combination that belonged to a list of pre-determined successful combinations. Low-level innovations (created by combining basic resources) could be combined to produce higher-level innovations. Further accumulation of innovations could produce complex tools (such as axes) that potentially allowed players to get logs (by cutting trees) to build their totem. From top to bottom: a ‘stick' could be obtained by placing a ‘bough' alone in the workshop. A ‘stone tool' could be obtained by combining two ‘refined stones'. A ‘string' could be obtained by combining two ‘bunches of fibres'. The combination of a bough, a string and a stone tool allowed players to get an ‘handaxe'. The handaxe allowed players to cut trees and get ‘logs'. The order of the items in the workshop panel had no effect on the result. (**c**) Examples of totem poles. Other high-level innovations (such as carving tools or pigments) could be subsequently used to refine totems to increase their value. Players' gain depended on the number of innovations they discovered and the value of their totem. The totem depicted on the left represents the achievement of the best isolated individual, required 22 innovations and scored 920 points. The totem depicted on the right represents the achievement of the best social learner, required 54 innovations and scored 6,526 points.

Individuals in groups produced much better results than isolated individuals. Only 18% of isolated individuals managed to build a totem, while 87% of participants from groups of six fully connected players with full information (full information treatment) were able to do it. Players from the full information treatment also produced much more complex and rewarding totems (that is, resulting from a building process that involved more innovations, Fig. 1c), and, on average, outperformed isolated individuals by a factor of 8 (mean=181, s.d.=222 and *M*=1,461, s.d.=1,599, respectively, Fig. 3). The best isolated individual (out of 60) that played our game scored 1,250, while 70% of people participating in the full information treatment achieved a score that was at least this high.

### Reasoning abilities

No isolated bot built a totem, and only 0.004% of all bots benefiting from social information were able to build a totem. The performances of bots and humans were affected by reasoning and social learning abilities, and the interaction between these factors (Fig. 3). As a consequence, isolated bots scored almost four times lower than isolated humans (*M*=51, s.d.=1), while groups of bots scored 15 times lower than groups of humans (*M*=99, s.d.=28).

### Social learning mechanisms

Participants who were provided with information about innovations, but not about the related production processes (partial information treatment) were able to build a totem 65% of time. They achieved scores that were three times higher than that of isolated individuals and about half of that achieved by players provided with full information (*M*=654, s.d.=500, Fig. 4). No individual in the partial information treatment was able to outperform the very best isolated individual. Thus, the cumulative cultural process broke down when individuals only had access to partial social information.

### Population structure

The success of participants in groups of three was intermediate between isolated players and players in groups of six. Players in groups of three with full social information managed to build a totem 70% of time and outperformed isolated players by a factor of 4 (*M*=774, s.d.=511, Fig. 5). None of the participants in groups of three were able to outperform the best isolated individual.

Participants in the partial connectivity treatment achieved scores that were quite similar to those of players in the full treatment (*M*=1275, s.d.=162). 90% of participants in the partial connectivity treatment earned scores that were as high as the most successful isolated individual and 100% of them were able to build a totem.

## Discussion

Our experiment provides the first empirical evidence that people working in groups are able to accumulate information and develop artefacts that are too complex for any isolated individual to invent during the same period. This result is consistent with the cultural niche hypothesis that holds that human populations would have been able to settle and prosper in harsh environments as a result of cultural information accumulation rather than evolved cognitive abilities alone^1^.

The comparison with bots, however, highlights the role of guided variation in the innovation production process. Even isolated humans outperformed groups of bots by a factor of 2. This striking difference is partially explained by the fact that bots searched for solutions at random, while evolved cognitive abilities allow humans to produce mental simulations that bias the way they explore their environments^19^. For example, in the first trial, the 60 isolated individuals produced only 20 different combinations (among 209 possibilities). Although all of these initial solutions were unsuccessful, this illustrates how humans provided only with an overall objective are able to reason about (1) what subordinate objectives could lead them to fulfil their ultimate goal and (2) which combinations are likely to be useful given the properties of the items involved. Furthermore, our results also suggest that individuals were able to generalize the function/properties of specific items within the game^20^, as indicated by the fact that the isolated players' average rate of successful combinations increased across time (likelihood ratio test (LRT): *χ*^2^=5.38, d.f.=1, *P*=0.02, *N*=431).

The humans' ability to produce successful guided variation was greatly amplified when it was combined with social information and allowed groups of six humans to score 15 times higher than same-sized groups of bots. This interaction between reasoning and social learning abilities could indicate that observing other players' solutions allowed social learners to refine their own intuitions about the properties of items faster than individuals working alone^21^. It is also possible that groups, due to complementary knowledge or experience, collectively explored a wider range of possibilities than individuals working alone, even though participants shared strong intuitions about which combinations are most likely to be successful. Consistent with this view, our results indicate that, on average, 5 out of 6 players produced at least one innovation that was not previously present within their own group, which might suggest that within-group interindividual variability could help explain the success of human groups^22^.

Even if the isolated players' average rate of successful combinations was low (2%), it is likely that the difference we observed between bots and humans was at least partially due to the experimental task we designed. Although our task was virtual, it was likely to elicit naive physics-based heuristics, which could have helped participants to produce successful combinations. Thus, we might expect the observed difference to be smaller if participants face a more demanding or causally opaque task (for example, kayak production)^23^.

Our third experiment demonstrates that social learning mechanisms strongly impact cultural accumulation. It is commonly acknowledged that the cultural evolution process requires information to be efficiently transmitted across generations, but the minimal social learning mechanism that allows information to accumulate across time is still widely debated^14^,^24^,^25^,^26^,^27^. Human social learning mechanisms, for example, seem more oriented towards production processes than those of chimpanzees^24^, and this could prevent information loss and promote cumulative culture. Experimental studies, comparing the performance of humans benefiting from product information with those benefiting from process information have produced mixed results^14^,^26^,^27^, demonstrating that the amount of information loss depends on the complexity of the task. Here we do not claim that the difference between the full information and partial information treatments illustrates the effects of process- and product-oriented learning mechanisms. Rather, we stress the fact that players faced transparent tasks in the full information treatment, and opaque ones in the partial information treatment. Players in the full information treatment could easily reproduce the targeted items because they were able to learn the required combination of resources, while players in the partial information treatment had to infer the missing combinations. Although process information made the tasks transparent in our experiment, ecologically valid tasks, such as stone tool production, may require still more evolved social learning mechanisms to be similarly transparent. For instance, even the acquisition of relatively simple Oldowan tool-making skills is greatly enhanced by verbal teaching^28^. More generally, one should expect more complex social learning mechanisms to be useful whenever these mechanisms allow individuals to acquire crucial information that cannot be easily inferred from process observation. Thus, the present experiment does not allow us to determine the minimum social learning mechanisms necessary for cultural accumulation. However, it does show that removing process information has a critical effect on cultural evolution. Compared with previous studies, our experiment most likely shows a more general effect of product and process-oriented social learning mechanisms because players from the partial information treatment had to successively infer the underlying combination of many different innovations (such stone tools, axes and so on) rather than the underlying production process of a single item^14^,^26^,^27^. Thus, the current results indicate that cumulative cultural evolution requires process-oriented social learning mechanisms, although some forms of teaching could also be necessary.

Our last experiment shows that changes in social structure might have had critical effects on the rise of cumulative culture. In addition to confirming recent experimental results about the effect of group size on cultural complexity^12^,^13^,^15^, our results suggest that the rate of cultural accumulation may be highest in sizable and partially connected populations (as indicated by an higher probability of building a totem). This counter intuitive result is most likely due to the fact that less efficient networks can lead to more thorough exploration of design space^29^ (but see ref. 30). In our game, players could produce different innovations from the same pool of items. This means that innovations that might have been discovered from a given pool of items were increasingly harder to find each time a new item was added to this pool due to combinatorial explosion. In the partial connectivity treatment, partially independent subgroups were more likely to discover all the innovations that a specific pool of items provide, before sharing them. The fact that reducing connectivity within six-player groups had no significant effect on average performances (and even some positive effects on exploration) could be especially important for explaining the burst of cultural complexity that occurred during the Upper Palaeolithic transition. Hunter-gatherer societies display a unique social structure involving extensive interaction between people living in different residential groups^31^,^32^. These results suggest that a change in ancestral interaction patterns that created larger, but only partially connected, interaction networks, could have elicited a burst of cultural complexity. Second, this kind of metapopulation structure could have an unsuspected effect on the innovation rate. Indeed, it has been suggested that partially connected networks are better at maintaining cultural diversity in multi-modal fitness landscapes^29^,^33^. As a consequence, recombination of pre-existing cultural traits, which some have argued is the main mode of technological innovation^34^, might have been greatly favoured within partially connected populations.

Our results are consistent with the hypothesis that cultural information accumulation played an essential role in the evolution of modern humans^1^. Although the present experiment involved bots generating random combinations, and do not replicate the actual mode of reasoning of a distant ancestor, our results illustrate how changes in the ability to produce successful guided variation may have speeded up innovation rates. According to previous theoretical work, an increase in reasoning abilities might have coevolved with more efficient and cognitively costly social learning abilities^6^,^35^, establishing propitious conditions for cultural information accumulation. Finally, our experiment confirms the critical role of demographic processes in cumulative cultural evolution, and suggests that shifts in ancestral between-group interaction patterns might have paved the way to behavioural modernity.

## Methods

### Participants

A total of 300 Arizona State University students (150 women and 150 men) were randomly selected from a database managed by the Elinor Ostrom Multi-Method Lab at Arizona State University and recruited by email. Informed consent was obtained from all subjects before starting the experiment (ethical approval was given by Arizona State University IRB, codes: STUDY00002137 and STUDY00002273). The subjects ranged in age from 18 to 26 years (mean 19 years, s.d. 1.37 years). Participants received $5 for participating and an additional amount ranging from $5 to $30 depending on their own performance.

### Procedure

The experiments took place in a computer room at the Elinor Ostrom Multi-Method Lab at Arizona State University. For each session, a maximum of 20 participants (exclusively male or female) were recruited and randomly assigned to one condition of the experiment. Participants sat at physically separated and networked computers and were randomly assigned to a group or worked alone. Players did not know who belonged to their group and were instructed that communication and note taking were not allowed. Before starting the experiment, participants were requested to enter their age and sex. Participants could read instructions on their screens. The game lasted 45 min, after which subjects received a reward according to their performance ($15 on average).

### Game principle

**Figure 2:**
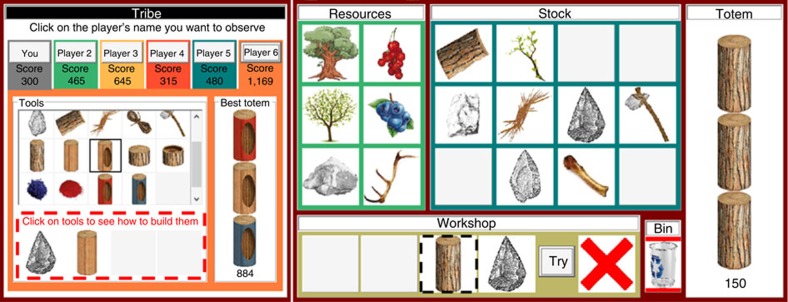
Game interface. Resources could be dropped into the ‘workshop panel' to be refined. Players could trigger an automatic refining process by clicking on the ‘try' button. A red cross indicated that the current combination was unsuccessful. Successful combinations resulted in a new item that could be dropped into the ‘stock panel' or in the ‘workshop panel' to be further refined. Logs (for example, the most basic log outlined in the black dashed square) were the minimal elements that could be dropped into the ‘totem panel' and provided players with a totem score. The ‘tribe panel' provided players with social information. The ‘tribe panel' depicted here illustrates the ‘full treatment' in which players benefited from five constant sources of information. By clicking onto an anonymised name, players could see the innovation record of the corresponding player. By clicking onto an item (for example, the carved log outlined in black), players could benefit from the underlying combination that resulted in this item (depicted in the red dashed rectangle). Players from the partial information treatment did not benefit from the information depicted in the red dashed rectangle. Players from the small group treatment benefited only from two constant sources of information. Players from the low connectivity treatment benefited only from one changing source of information (among five). Isolated players benefited from the same panel (relabelled ‘innovation record') but were not able to switch between their own record and any other players' record. All players benefited from underlying combinations when clicking on their own innovations.

**Figure 3:**
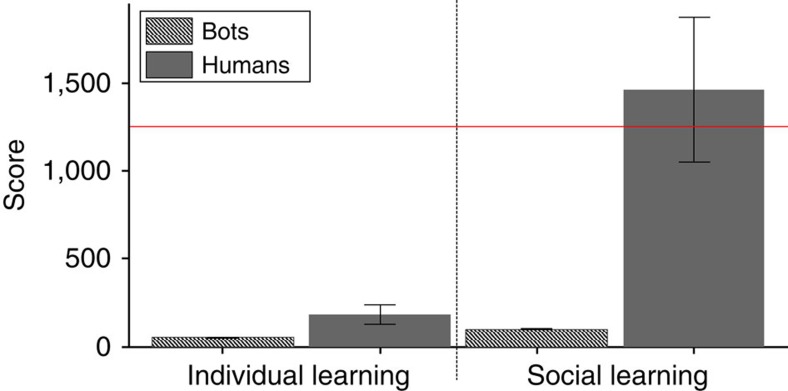
Reasoning abilities and social learning affect the information accumulation. The final achievements of individuals were significantly affected by reasoning abilities (bots versus humans, likelihood ratio test (LRT): *χ*^2^=534, d.f.=1, *P*<0.001, *N*=2120), social learning (LRT: *χ*^2^=1374, d.f.=1, *P*<0.001, *N*=2120) and the interaction between both (LRT: *χ*^2^=159, d.f.=1, *P*<0.001, *N*=2120). The horizontal red line shows the score of the very best isolated individual, which was equated or outperformed by 70% of participants from the full treatment (full dark grey bar). Both human-based treatments involved 60 participants. Simulations involving bots were run 1,000 times. The error bars show 95% confidence intervals.

**Figure 4:**
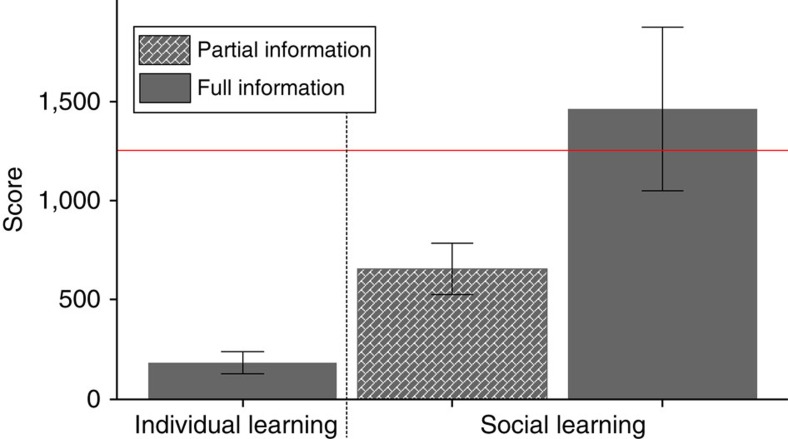
Cumulative culture depends on social learning mechanisms that make information easy to learn. Players from the full information treatment were provided with the underlying combination when clicking on other players' innovations, while players from the partial information did not. Both full and partial social information allowed players to outperform individual learners (LRT: *χ*^2^=29.3, d.f.=1, *P*<0.001, *N*=120 and *χ*^2^=24.7, d.f.=1, *P*<0.001, *N*=120, respectively), but only some players from the former treatment scored higher than the best isolated individual (represented by the horizontal red line). On average, players benefiting from the full social information outperformed those who benefited only from partial social information (LRT: *χ*^2^=4.59, d.f.=1, *P*<0.04, *N*=120). All treatments involved 60 participants. The error bars show 95% confidence intervals.

**Figure 5:**
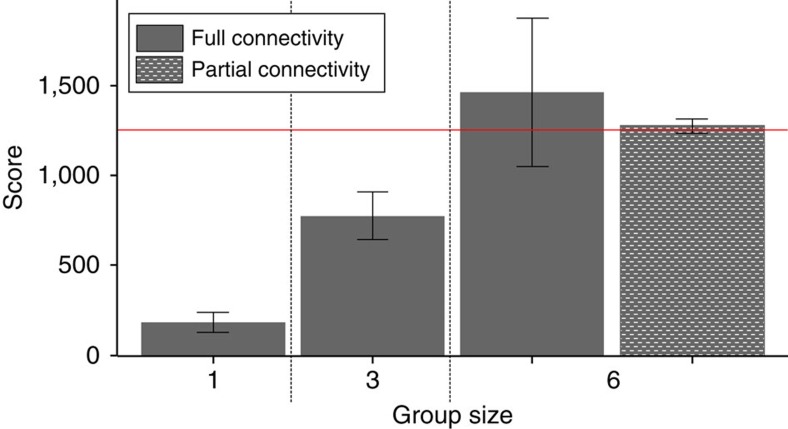
Population structure affects cultural accumulation. Group size had a significant linear effect on the performance of players (LRT: *χ*^2^=37.9, d.f.=1, *P*<0.001, *N*=180). Players from the partial connectivity treatment, who experienced a 3 × 2-player metapopulation structure, outperformed individual learners (LRT: *χ*^2^=41.2, d.f.=1, *P*<0.001, *N*=120) and did not significantly differ from players from fully connected six-player groups (LRT: χ^2^=0.90, d.f.=1, *P*=0.34, *N*=120). The horizontal red line shows the score of the best isolated individual. Players from the partial connectivity treatment equated or outperformed the score of the best isolated individual in 90% of cases. All treatments involved 60 participants. The error bars show 95% confidence intervals.

The participants played a computer game (programmed in Object Pascal with Delphi 6) that simulated a real-world innovation process in which the production of complex artefacts depended on the discovery of high-level innovations. Discovering these innovations was contingent on the discovery of lower-level innovations. Both low- and high-level innovations resulted from a specific production process that was initially unknown to participants. Players were initially provided with six basic resources (Fig. 1a) that could be used without any limit and combined using a workshop panel containing four slots (Fig. 2). After dropping between one and four resources into this panel, players could trigger an automatic refining process at no cost and without any limit by clicking on a ‘Try' button. Innovations arose when players produced a combination that belonged to a list of pre-determined successful combinations (Fig. 1b). A specific slot displayed the result: a red cross when the combination was unsuccessful, a new item otherwise. When discovered, new items could be in turn associated with other items to produce higher-level innovations. All combinations were allowed, including those involving the repeated use of the same item. The order of the items in the workshop panel had no effect on the result, so that 209 unique combinations could be produced from six initial resources. The production of new items led to a combinatorial explosion, so that 1,000 different combinations could be produced after the discovering of four new items/innovations. In total, 27 additional items (all useful) could be generated from the six initial resources. A stock panel allowed the players to store up to 12 items, in addition to the six initial resources (Fig. 2). The accumulation of innovations could result in the production of complex tools (such as axes) that potentially allowed players to get logs by cutting trees. Basic logs required at least eight innovations to be produced and were the minimal element that could be dropped into a three-slot totem pole panel, which provided players with a totem score. Logs could be refined when combined with relevant tools (such as carving tools, pigments, brushes and so on) in the workshop panel. 115 different logs could be produced, so that a total of 142 innovations and 266,915 unique totems could be generated.

### Tutorial and pre-game information

Before starting, the players had to complete a tutorial during which basic actions, such as dragging and dropping resources into the workshop panel, had to be completed. The tutorial also guided player's actions until the production of a first innovation (the same one for all the players) to make sure that all players mastered the game interface before starting the experiment. Players were informed that the ultimate aim of the game was to build a totem pole, that innovations had to be produced before being able to produce logs and that these logs could be used and refined to make totems. Players had no idea about which items could be produced during the game. Players were also informed that their score, and their monetary reward depended on the number of new items they were able to produce and the value of their totem. The fitness function that determined the value of a totem was unknown to players.

### Score calculation

Each of the 115 different logs was associated with a unique value that was randomly attributed within a range of scores that depended on the log's complexity. The complexity of logs was defined by the number of innovations that was required to produce them. It means that logs with higher number of underlying innovations were always more rewarding, although two logs with the same number of underlying innovations did not have the same value. The score of a totem, which depended on the value of the logs and their diversity, was calculated as follow:


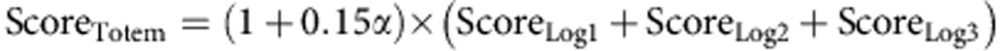


With *α* taking the value 0, 1 or 2 depending on whether the totem pole involved 1, 2 or 3 different logs.

Totem scores ranged from 50 to 7,410 points. The players' final score was equal to the score of the best totem they built plus 15 points for each new item they produced.

### Treatments

All players were provided with an additional panel whose content varied according to the treatment (Fig. 2). Players from the individual learning treatment were only provided with their own score and a record of their own innovations (alongside their best totem, if any). All players could click on innovations from their own record to generate a reminder about how to produce them. Players from other treatments benefited from additional information and could switch between their own record and others' record by clicking on an anonymised name (such as ‘player 3') and associated score (Fig. 2). Other players' scores were updated every 10 s. Players from the full and partial information treatments were permanently provided with five constant sources of social information. However, participants in the full information treatment were provided with the underlying combination when they clicked on other players' innovations, while participants in the partial information treatment were not allowed to see the underlying combination (Fig. 2). Players from the small group and partial connectivity treatments benefited from the same innovation-related social information as in the full treatment, except that participants in the small group treatment benefited only from two constant sources of information (from the other members of their group), while the latter benefited from 1 changing source of information (among 5). In the partial connectivity treatment, the between-players ties were always reciprocal, so that, at any time, the population structure can be described as a 3 × 2-player group metapopulation. The between-players ties were randomly generated and varied every 3 min, so that, on average, the probability of connecting every possible pairs of individuals during the course of the experiment was about 1. Players provided with social information could observe other group members without any limit. All treatments involved 30 men and 30 women in single-sex groups (to facilitate statistical analyses).

### Bots

Isolated bots generated combinations through a two-step process: they first choose a random number *N* of workshop slots (1≤*N*≤4) before randomly selecting *N* items from the same initial pool of items as human participants. When they generated a successful combination, the resulting innovation was added to their pool of items, which was used during subsequent trials. The number of attempts performed by bots was parametrized using data generated by humans. Human participants produced an average of 380 attempts, but half of them were redundant (51%), which is mainly explained by a limited memory capacity (an average 45% of unsuccessful combinations were redundant combinations). As we were interested in the effect of the ability to generate guided variation (and not in the effect of memory), we allowed bots to generate 188 unique attempts/combinations (that is, new combinations were generated at no cost when bots randomly produced an already tried combination), which was the average number of unique combinations that human participants produced during our experiment (the results are not sensitive to variation in the number of attempts around this mean). The final score of isolated bots was based on the number of innovations they discovered as no isolated bots were able to produce logs (the minimal element required to build a totem). Groups of six bots generated combinations according to the same process, except that they benefited from the innovations of other bots in addition to their own discovery, which simulated the effect of social learning. Bots could instantly use other bots' discoveries to generate new combinations and progress further in the fitness landscape (as each innovation was useful). Bots that produced logs were provided with a totem score that equalled the maximum number of points that could be obtained from these logs. The social bots final score was based on the number of innovations they produced and their totem score.

### Analyses

All statistical analyses of scores were based on linear mixed models with the log-transformed individuals' final score as the response variable and group identity as a random effect. Preliminary analyses revealed no effect of sex or age and were not introduced in the final statistical models.

*Reasoning abilities and social learning*. The data set was composed of the performance of humans and bots that were either isolated or organized in groups of six. The binary variables ‘reasoning abilities' (that is humans or bots) and ‘social learning' and the interaction between both were evaluated as explanatory variables.

*Social learning mechanisms*. The data associated with the ‘isolated individuals', ‘partial information' and ‘full information' treatments were considered. ‘Treatment' was introduced as explanatory variable.

*Population structure*. Two analyses were run. In the first, the data set was composed of the data from the ‘isolated individuals', ‘small group' (three-player group) and ‘full' (six-player groups) treatments, and ‘group size' was introduced in the model as a continuous variable; in the other, the data set was composed of the data from ‘partial connectivity' and ‘full connectivity' treatments, and ‘connectivity' was modelled as a binary variable (low or high).

*Rate of innovation*. To determine whether humans were able to generalize the function of specific items within the game, we investigated the number of unsuccessful combinations that isolated players had to generate before producing a successful one. This log-transformed value was the response variable. The rank of the innovation (within the player's own innovation record) was evaluated as an explanatory variable. Theoretically, the response variable should be strongly affected by the number of possible combinations that one player was able to generate from his/her pool of items. For this reason, we introduced the corresponding log-transformed number of possible combinations that the player could produce as a control variable. The player's identity was introduced as random variable. Our results indicate that the number of unsuccessful combinations that players had to generate before producing a successful one was negatively affected by the rank of the innovation (LRT: *χ*^2^=5.32, d.f.=1, *P*=0.02, *N*=431), indicating that players got better at generating successful combinations across time. As expected, the number of possible combinations negatively affected the individuals' ability to find successful combinations, although this effect was only marginally significant (LRT: *χ*^2^=3.02, d.f.=1, *P*=0.08, *N*=431).

All statistical analyses were conducted using R version 3.0.1 (ref. 36). The significance of explanatory variables was assessed by comparing full and restricted models using LRTs and parametric bootstrapping with 1,000 simulations. Both tests yielded qualitatively similar results. Mixed models, LRTs and parametric bootstrapping were performed using the lme4 (ref. 37) and pbkrtest^38^ packages.

## Additional information

**How to cite this article:** Derex, M. *et al*. The foundations of the human cultural niche. *Nat. Commun.* 6:8398 doi: 10.1038/ncomms9398 (2015).
